# 2-Cyano-*N*′-(5-hy­droxy-2-nitro­benzyl­idene)acetohydrazide monohydrate

**DOI:** 10.1107/S1600536811025463

**Published:** 2011-07-09

**Authors:** Hongbo Li, Xiaocheng Ni

**Affiliations:** a College of Chemistry and Biology Engineering, Yancheng Institute of Technology, Yancheng 224051, People’s Republic of China

## Abstract

The title compound, C_10_H_8_N_4_O_4_·H_2_O, was obtained by the reaction of 5-hy­droxy-2-nitro­benzaldehyde with cyano­acetohydrazide in methanol. The non-H atoms of the hydrazone molecule are approximately coplanar, with a mean deviation from the least-squares plane of 0.056 Å. In the crystal, mol­ecules are linked by N—H⋯O, O—H⋯N and O—H⋯O hydrogen bonds, generating a three-dimensional network.

## Related literature

For the structures of hydrazones, see: Wang *et al.* (2011 ▶); Hashemian *et al.* (2011 ▶); Singh & Singh (2010 ▶); Ahmad *et al.* (2010 ▶).
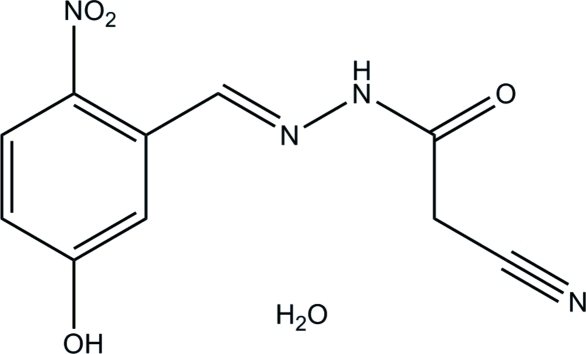


## Experimental

### 

#### Crystal data

C_10_H_8_N_4_O_4_·H_2_O
*M* *_r_* = 266.22
Monoclinic, 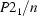

*a* = 4.663 (1) Å
*b* = 13.238 (2) Å
*c* = 19.305 (2) Å
β = 90.312 (3)°
*V* = 1191.7 (3) Å^3^
*Z* = 4
Mo *K*α radiation
μ = 0.12 mm^−1^
*T* = 298 K
0.27 × 0.23 × 0.23 mm

#### Data collection

Bruker SMART 1K CCD area-detector diffractometer
Absorption correction: multi-scan (*SADABS*; Sheldrick, 2004 ▶) *T*_min_ = 0.968, *T*_max_ = 0.973
8851 measured reflections
2531 independent reflections
1935 reflections with *I* > 2σ(*I*)
*R*_int_ = 0.028

#### Refinement

*R*[*F* ^2^ > 2σ(*F* ^2^)] = 0.054
*wR*(*F* ^2^) = 0.148
*S* = 1.04
2531 reflections
184 parameters
5 restraints
H atoms treated by a mixture of independent and constrained refinement
Δρ_max_ = 0.43 e Å^−3^
Δρ_min_ = −0.20 e Å^−3^

### 

Data collection: *SMART* (Bruker, 2001 ▶); cell refinement: *SAINT* (Bruker, 2001 ▶); data reduction: *SAINT*; program(s) used to solve structure: *SHELXTL* (Sheldrick, 2008 ▶); program(s) used to refine structure: *SHELXTL*; molecular graphics: *SHELXTL*; software used to prepare material for publication: *SHELXTL*.

## Supplementary Material

Crystal structure: contains datablock(s) I, global. DOI: 10.1107/S1600536811025463/qm2014sup1.cif

Structure factors: contains datablock(s) I. DOI: 10.1107/S1600536811025463/qm2014Isup2.hkl

Supplementary material file. DOI: 10.1107/S1600536811025463/qm2014Isup3.cml

Additional supplementary materials:  crystallographic information; 3D view; checkCIF report

## Figures and Tables

**Table 1:** Hydrogen-bond geometry (Å, °)

*D*—H⋯*A*	*D*—H	H⋯*A*	*D*⋯*A*	*D*—H⋯*A*
O5—H5*B*⋯N4^i^	0.84 (1)	2.32 (2)	3.117 (4)	158 (3)
O5—H5*A*⋯O1^ii^	0.84 (1)	2.22 (2)	3.017 (3)	157 (3)
O4—H4⋯O5	0.86 (1)	1.85 (1)	2.700 (3)	170 (3)
N3—H3*A*⋯O3^iii^	0.90 (1)	1.98 (1)	2.880 (2)	177 (2)

Symmetry codes: (i) 
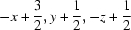
; (ii) 
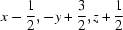
; (iii) 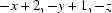.

**Fig. 1.**
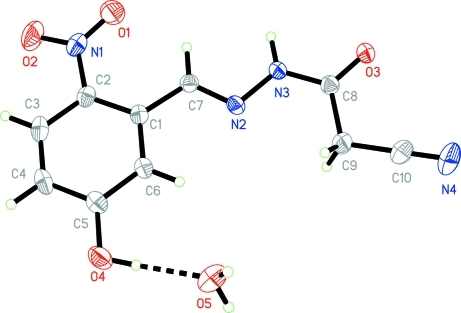
The molecular structure of (I), with atom labels and 30% probability displacement ellipsoids for non-H atoms. O—H···O hydrogen bond is shown as a dashed line.

**Fig. 2.**
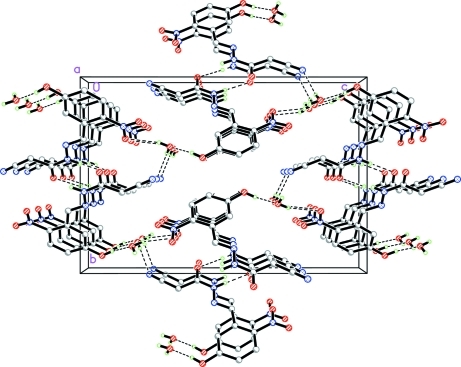
The packing of (I), viewed down the a axis. Hydrogen bonds are shown as dashed lines. H atoms not involved in hydrogen bonding have been omitted.

## supplementary crystallographic information

### Comment

Recently, a great number of hydrazones derived from the reaction of benzaldehyde and its derivatives with benzohydrazides have been reported (Wang *et al.*, 2011; Hashemian *et al.*, 2011; Singh & Singh, 2010; Ahmad *et al.*, 2010). To the best of our knowledge, the hydrazones derived from cyanoacetohydrazide have never been reported so far. In this paper, the title new hydrazone compound, (I), is reported.

The compound contains a hydrazone molecule and a water molecule (Fig. 1). The non-hydrogen atoms of the hydrazone molecule are approximately coplanar, with mean deviation from the least-squares plane of 0.056 (3) Å. In the crystal structure, molecules are linked by intermolecular N—H···O, O—H···O, and O—H···N hydrogen bonds (Table 1), generating a three-dimensional network (Fig. 2).

### Experimental

The title compound was obtained by the reaction of equimolar quantities (1.0 mmol each) of 5-hydroxy-2-nitrobenzaldehyde with cyanoacetohydrazide in methanol. Single crystals suitable for X-ray diffraction were obtained by the slow evaporation of the solution containing the compound in open air.

### Refinement

H atoms bonded to N3, O4 and O5 atoms were located in a difference map and refined with distance restraints of O—H = 0.85 (1) Å, N—H = 0.90 (1) Å, and with *U*_iso_(H) = 1.5*U*_eq_(O) and 1.2*U*_eq_(N). Other H atoms were positioned geometrically and refined using a riding model, with C—H = 0.93–0.97 Å, and with *U*_iso_(H) = 1.2*U*_eq_(C).

### Crystal data

**Table:**

C_10_H_8_N_4_O_4_·H_2_O	*F*(000) = 552
*M**_r_* = 266.22	*D*_x_ = 1.484 Mg m^−^^3^
Monoclinic, *P*2_1_/*n*	Mo *K*α radiation, λ = 0.71073 Å
*a* = 4.663 (1) Å	Cell parameters from 2122 reflections
*b* = 13.238 (2) Å	θ = 2.6–26.6°
*c* = 19.305 (2) Å	µ = 0.12 mm^−^^1^
β = 90.312 (3)°	*T* = 298 K
*V* = 1191.7 (3) Å^3^	Block, colorless
*Z* = 4	0.27 × 0.23 × 0.23 mm

### Data collection

**Table:**

Bruker SMART 1K CCD area-detector diffractometer	2531 independent reflections
Radiation source: fine-focus sealed tube	1935 reflections with *I* > 2σ(*I*)
graphite	*R*_int_ = 0.028
ω scans	θ_max_ = 27.0°, θ_min_ = 1.9°
Absorption correction: multi-scan (*SADABS*; Sheldrick, 2004)	*h* = −5→5
*T*_min_ = 0.968, *T*_max_ = 0.973	*k* = −16→16
8851 measured reflections	*l* = −24→23

### Refinement

**Table:**

Refinement on *F*^2^	Primary atom site location: structure-invariant direct methods
Least-squares matrix: full	Secondary atom site location: difference Fourier map
*R*[*F*^2^ > 2σ(*F*^2^)] = 0.054	Hydrogen site location: inferred from neighbouring sites
*wR*(*F*^2^) = 0.148	H atoms treated by a mixture of independent and constrained refinement
*S* = 1.04	*w* = 1/[σ^2^(*F*_o_^2^) + (0.0727*P*)^2^ + 0.3422*P*] where *P* = (*F*_o_^2^ + 2*F*_c_^2^)/3
2531 reflections	(Δ/σ)_max_ = 0.001
184 parameters	Δρ_max_ = 0.43 e Å^−^^3^
5 restraints	Δρ_min_ = −0.20 e Å^−^^3^

### Special details

**Table:**

Geometry. All e.s.d.'s (except the e.s.d. in the dihedral angle between two l.s. planes) are estimated using the full covariance matrix. The cell e.s.d.'s are taken into account individually in the estimation of e.s.d.'s in distances, angles and torsion angles; correlations between e.s.d.'s in cell parameters are only used when they are defined by crystal symmetry. An approximate (isotropic) treatment of cell e.s.d.'s is used for estimating e.s.d.'s involving l.s. planes.
Refinement. Refinement of *F*^2^ against ALL reflections. The weighted *R*-factor *wR* and goodness of fit *S* are based on *F*^2^, conventional *R*-factors *R* are based on *F*, with *F* set to zero for negative *F*^2^. The threshold expression of *F*^2^ > σ(*F*^2^) is used only for calculating *R*-factors(gt) *etc*. and is not relevant to the choice of reflections for refinement. *R*-factors based on *F*^2^ are statistically about twice as large as those based on *F*, and *R*- factors based on ALL data will be even larger.

### Fractional atomic coordinates and isotropic or equivalent isotropic displacement parameters (Å^2^)

**Table:**

	*x*	*y*	*z*	*U*_iso_*/*U*_eq_	
N1	0.1870 (4)	0.73554 (14)	−0.16790 (9)	0.0507 (5)	
N2	0.5017 (3)	0.64853 (11)	0.03310 (8)	0.0366 (4)	
N3	0.7235 (3)	0.57994 (11)	0.03500 (8)	0.0369 (4)	
N4	0.9875 (6)	0.5194 (2)	0.26634 (12)	0.0898 (8)	
O1	0.3932 (4)	0.67914 (16)	−0.16953 (9)	0.0813 (6)	
O2	0.0556 (5)	0.75595 (16)	−0.22076 (9)	0.0877 (7)	
O3	1.0378 (3)	0.48653 (11)	0.09451 (7)	0.0476 (4)	
O4	−0.2421 (3)	0.91461 (12)	0.07021 (8)	0.0574 (4)	
O5	0.0680 (5)	0.8597 (2)	0.18259 (10)	0.0919 (7)	
C1	0.2056 (4)	0.75195 (13)	−0.03818 (9)	0.0350 (4)	
C2	0.0916 (4)	0.77958 (14)	−0.10307 (10)	0.0403 (5)	
C3	−0.1218 (5)	0.85279 (15)	−0.10828 (12)	0.0481 (5)	
H3	−0.1921	0.8711	−0.1517	0.058*	
C4	−0.2291 (5)	0.89799 (15)	−0.05048 (12)	0.0489 (5)	
H4A	−0.3714	0.9469	−0.0545	0.059*	
C5	−0.1244 (4)	0.87064 (14)	0.01452 (11)	0.0424 (5)	
C6	0.0926 (4)	0.79932 (13)	0.01966 (10)	0.0378 (4)	
H6	0.1646	0.7828	0.0632	0.045*	
C7	0.4343 (4)	0.67707 (13)	−0.02755 (9)	0.0362 (4)	
H7	0.5302	0.6505	−0.0655	0.043*	
C8	0.8365 (4)	0.54622 (14)	0.09432 (10)	0.0379 (4)	
C9	0.7002 (5)	0.58300 (19)	0.16020 (10)	0.0589 (6)	
H9A	0.6945	0.6562	0.1602	0.071*	
H9B	0.5047	0.5583	0.1628	0.071*	
C10	0.8611 (6)	0.54791 (19)	0.21982 (12)	0.0608 (6)	
H3A	0.804 (5)	0.5585 (18)	−0.0045 (8)	0.073*	
H4	−0.158 (6)	0.891 (2)	0.1062 (10)	0.091*	
H5A	−0.017 (5)	0.839 (2)	0.2181 (10)	0.091*	
H5B	0.220 (4)	0.889 (2)	0.1943 (14)	0.091*	

### Atomic displacement parameters (Å^2^)

**Table:**

	*U*^11^	*U*^22^	*U*^33^	*U*^12^	*U*^13^	*U*^23^
N1	0.0566 (12)	0.0555 (11)	0.0398 (10)	−0.0081 (9)	−0.0049 (8)	0.0066 (8)
N2	0.0351 (9)	0.0339 (8)	0.0408 (9)	0.0058 (6)	−0.0004 (7)	−0.0017 (6)
N3	0.0367 (9)	0.0388 (8)	0.0352 (8)	0.0084 (7)	0.0004 (6)	−0.0012 (6)
N4	0.1006 (19)	0.117 (2)	0.0512 (13)	0.0011 (16)	−0.0282 (13)	0.0015 (13)
O1	0.0820 (13)	0.1149 (16)	0.0470 (10)	0.0336 (12)	−0.0046 (9)	−0.0139 (10)
O2	0.1152 (17)	0.1065 (15)	0.0413 (10)	0.0179 (12)	−0.0188 (10)	0.0071 (9)
O3	0.0456 (8)	0.0548 (8)	0.0423 (8)	0.0165 (7)	−0.0015 (6)	0.0026 (6)
O4	0.0541 (10)	0.0521 (9)	0.0661 (11)	0.0165 (7)	0.0021 (8)	−0.0071 (8)
O5	0.1056 (17)	0.123 (2)	0.0469 (10)	0.0166 (14)	0.0030 (10)	0.0008 (11)
C1	0.0313 (10)	0.0330 (9)	0.0407 (10)	−0.0045 (7)	−0.0021 (8)	0.0031 (7)
C2	0.0410 (11)	0.0392 (10)	0.0406 (10)	−0.0081 (8)	−0.0041 (8)	0.0065 (8)
C3	0.0465 (12)	0.0432 (11)	0.0543 (12)	−0.0048 (9)	−0.0138 (10)	0.0141 (9)
C4	0.0405 (12)	0.0360 (10)	0.0700 (14)	0.0044 (8)	−0.0092 (10)	0.0097 (10)
C5	0.0377 (11)	0.0321 (9)	0.0575 (12)	−0.0025 (8)	−0.0001 (9)	−0.0012 (8)
C6	0.0341 (10)	0.0347 (9)	0.0447 (10)	0.0002 (8)	−0.0033 (8)	0.0031 (8)
C7	0.0356 (10)	0.0368 (9)	0.0362 (10)	0.0000 (8)	0.0014 (7)	0.0001 (7)
C8	0.0378 (11)	0.0378 (10)	0.0380 (10)	0.0020 (8)	−0.0010 (8)	−0.0015 (8)
C9	0.0643 (15)	0.0749 (16)	0.0375 (11)	0.0229 (12)	−0.0052 (10)	−0.0076 (10)
C10	0.0675 (16)	0.0742 (16)	0.0405 (12)	0.0013 (13)	−0.0063 (11)	−0.0082 (11)

### Geometric parameters (Å, °)

**Table:**

N1—O2	1.218 (2)	C1—C2	1.407 (3)
N1—O1	1.218 (2)	C1—C7	1.470 (2)
N1—C2	1.453 (3)	C2—C3	1.392 (3)
N2—C7	1.268 (2)	C3—C4	1.363 (3)
N2—N3	1.377 (2)	C3—H3	0.9300
N3—C8	1.335 (2)	C4—C5	1.392 (3)
N3—H3A	0.898 (10)	C4—H4A	0.9300
N4—C10	1.136 (3)	C5—C6	1.387 (3)
O3—C8	1.227 (2)	C6—H6	0.9300
O4—C5	1.343 (3)	C7—H7	0.9300
O4—H4	0.856 (10)	C8—C9	1.506 (3)
O5—H5A	0.840 (10)	C9—C10	1.447 (3)
O5—H5B	0.839 (10)	C9—H9A	0.9700
C1—C6	1.387 (3)	C9—H9B	0.9700
			
O2—N1—O1	120.5 (2)	C5—C4—H4A	120.2
O2—N1—C2	118.5 (2)	O4—C5—C6	122.62 (18)
O1—N1—C2	120.92 (17)	O4—C5—C4	117.80 (18)
C7—N2—N3	113.79 (15)	C6—C5—C4	119.59 (19)
C8—N3—N2	122.43 (15)	C1—C6—C5	122.01 (18)
C8—N3—H3A	117.2 (17)	C1—C6—H6	119.0
N2—N3—H3A	120.3 (17)	C5—C6—H6	119.0
C5—O4—H4	108 (2)	N2—C7—C1	120.39 (17)
H5A—O5—H5B	110 (2)	N2—C7—H7	119.8
C6—C1—C2	117.14 (17)	C1—C7—H7	119.8
C6—C1—C7	118.09 (16)	O3—C8—N3	121.08 (17)
C2—C1—C7	124.77 (17)	O3—C8—C9	122.14 (17)
C3—C2—C1	120.78 (19)	N3—C8—C9	116.76 (17)
C3—C2—N1	116.07 (18)	C10—C9—C8	110.41 (19)
C1—C2—N1	123.15 (18)	C10—C9—H9A	109.6
C4—C3—C2	120.77 (19)	C8—C9—H9A	109.6
C4—C3—H3	119.6	C10—C9—H9B	109.6
C2—C3—H3	119.6	C8—C9—H9B	109.6
C3—C4—C5	119.69 (19)	H9A—C9—H9B	108.1
C3—C4—H4A	120.2	N4—C10—C9	179.3 (3)

### Hydrogen-bond geometry (Å, °)

**Table:**

*D*—H···*A*	*D*—H	H···*A*	*D*···*A*	*D*—H···*A*
O5—H5B···N4^i^	0.84 (1)	2.32 (2)	3.117 (4)	158 (3)
O5—H5A···O1^ii^	0.84 (1)	2.22 (2)	3.017 (3)	157 (3)
O4—H4···O5	0.86 (1)	1.85 (1)	2.700 (3)	170 (3)
N3—H3A···O3^iii^	0.90 (1)	1.98 (1)	2.880 (2)	177 (2)

Symmetry codes: (i) −*x*+3/2, *y*+1/2, −*z*+1/2; (ii) *x*−1/2, −*y*+3/2, *z*+1/2; (iii) −*x*+2, −*y*+1, −*z*.
